# Discovery of novel TLR modulators by Molecular Modeling and Virtual Screening

**DOI:** 10.1186/1758-2946-4-S1-P58

**Published:** 2012-05-01

**Authors:** Manuela S Murgueitio, Sandra Santos-Sierra, Gerhard Wolber

**Affiliations:** 1 Pharmaceutical Chemistry, Institute of Pharmacy, Freie Universität Berlin, Berlin, Berlin,14195, Germany; 2 Institute of Biochemical Pharmacology, Medizinische Universität Innsbruck, A-6020, Austria

## 

Toll-like receptors (TLRs) play a crucial role in the onset of innate immunity by distinguishing between endogenous and pathogen-associated molecular patterns. TLR2, in cooperation with TLR1 and TLR6, recognizes several microbial components such as lipoteichoic acids and lipoproteins [1]. Toll-like receptors have been broadly reported to contribute to several inflammatory chronic diseases and autoimmune diseases [2]. In this study we aim to discover new TLR2 modulating agents through computer-aided drug design.

Based on recently identified synthetic TLR2 agonists [3] and antagonists [4], a shape and chemical-feature based similarity search was performed against a library of 260.071 compounds provided by the National Cancer Institute (NCI) [5]. This led to several virtual hits, which were tested *in vitro* in a cell-based assay. Several compounds with biological activity on TLR2 signaling in general and TLR1 signaling specifically were identified.

To further optimize these biologically validated virtual hits, molecular interaction fields (MIFs) for the dimerization of TLR2 and TLR1 were developed. Feature-based MIFs allowed for the manual creation of virtual compounds that fulfill an optimized interaction pattern, which led to a 3D pharmacophore that was used for a second virtual screening to select compounds for biological testing.

## References

[B1] Akira S Takeda K Toll-like Receptor Signalling Nat Rev Immunol 2004 4 7 499 511 10.1038/nri1391 15229469

[B2] Kawai T Akira S The role of pattern-recognition receptors in innate immunity: update on Toll-like receptors Nat Rev Immunol 2010 11 5 373 384 10.1038/ni.1863 20404851

[B3] Guan Y Identification of Novel Synthetic Toll-like Receptor 2 Agonists by High Throughput Screening J Biol Chem 2010 258 31 23755 23762 20504771 10.1074/jbc.M110.116046 PMC2911294

[B4] Zhou S Discovery of a novel TLR2 signaling inhibitor with anti-viral activity Antiviral Res 2010 87 3 295 306 10.1016/j.antiviral.2010.06.011 20603154 PMC2924919

[B5] http://cactus.nci.nih.gov/download/nci/

